# Reply to Alonso et al. “Bangladesh and Rwanda: Cases of high burden of influenza in tropical countries?”

**DOI:** 10.1111/irv.12576

**Published:** 2018-06-17

**Authors:** Makhdum Ahmed, Katherine Roguski, Stefano Tempia, Angela D. Iuliano

**Affiliations:** ^1^ International Centre for Diarrheal Disease Research Dhaka Bangladesh; ^2^ Department of Lymphoma and Myeloma The University of Texas MD Anderson Cancer Center Houston TX USA; ^3^ Influenza Division Centers for Disease Control and Prevention Atlanta GA USA

## DISCLAIMER

The findings and conclusions in this report are those of the authors and do not necessarily represent the official position of the Centers for Disease Control and Prevention.

Dear Editor,

We thank Dr. Alonso et al for their commentary^1^ on our articles, “Estimates of Seasonal Influenza‐Associated Mortality in Bangladesh, 2010‐2012”^2^ and “The National Burden of Influenza‐Associated Severe Acute Respiratory Illness Hospitalization in Rwanda, 2012‐2014.”^3^ In their commentary, they described three assumptions that we would like to address: (1) their use of “substantial” burden compared to “high” burden, (2) the comparability of influenza burden in tropical climate countries, and (3) the impact of the influenza A(H1N1)pdm09 virus on mortality. In addition, they describe three concerns about our estimates, which we would also like to clarify, specifically: (4) a mismatch in the timing of respiratory deaths and the influenza virus circulation period, (5) mortality attribution, and (6) the comparison with Institute of Health Metrics and Evaluation (IHME) estimates. We will address each of these comments or concerns in this brief response.

1. “Substantial” vs “high” burden

While we noted that there is substantial burden from influenza virus infection in Bangladesh in 2010‐2012,^2^ our estimates are not higher than other estimates published from neighboring countries (reviewed in detail here^4^). Estimates from Kenya,^5^ South Africa,^6^ and Thailand^7^ are comparable to our estimates, and in some instances, higher than estimates for Bangladesh. As such, we do not consider the mortality estimates (6‐11 per 100 000 population) in Bangladesh to be high when compared to other countries; however, we do consider 6‐11 deaths per 100 000 population to be a substantial burden for the country of Bangladesh. Similarly, the estimated influenza‐associated hospitalization rates in Rwanda (34 per 100 000 population) are comparable to those of other African countries situated in equatorial, tropical, and subtropical regions: 21 (95% CI: 19‐23) per 100 000 population in Kenya,^8^ 30 (95% CI: 13‐84) per 100 000 population in Ghana,^9^ 30 (95% CI: 24‐36) per 100 000 population in Madagascar,^10^ 45 (95% CI: 41‐49) per 100 000 population in South Africa,^11^ and 44 (95% CI: 31‐57) per 100 000 population in Zambia.^12^ In addition, a global study reported the highest rates of influenza‐associated hospitalization among African children followed by South‐East Asia and the Western Pacific.^13^ Given the estimated burden in these countries, it seems reasonable for policymakers to explore the relative value of influenza prevention and control strategies among these populations.

- Influenza burden in tropical climate countries

The authors made general comparisons between tropical and temperate climate countries and indicated that there is a lower burden of influenza disease in tropical climate countries. However, we are not convinced that this comparison is generalizable to all tropical countries. For instance, not all tropical countries have year‐round influenza virus circulation as the authors suggest, including Bangladesh and Rwanda.^14^ Bangladesh has a distinct peak in influenza virus circulation during the monsoon season from April to September each year,^15^ whereas Rwanda experiences increased influenza virus circulation during the long rainy season from January to July.^16^ Furthermore, differences in underlying population health status, health systems, and age structure should also be considered when comparing burden estimates from different countries, as these factors can affect burden. The authors noted that Brazil has a homogenous public health system with immunization policies. Bangladesh and Rwanda do not have similar health systems, and there are currently no immunization policies for seasonal influenza vaccine in either country. Additionally, the population density of Bangladesh is among the highest in the world (1252 people per square kilometer in Bangladesh) and Rwanda has the second highest population density in Africa (440 people per square kilometer) compared with Brazil, which has one of the lowest population densities among all countries of the world (25 people per square kilometer).^17^ Population density may influence influenza virus transmission dynamics and burden of influenza virus infections.^18^ Given the important differences in public health systems, and sociodemographic factors between Brazil, Bangladesh, and Rwanda, disparities in the influenza virus disease burden in these countries are entirely possible. A comparative study of influenza disease burden between countries accounting for these differences would be valuable to better understand the effect of influenza virus infection on these different populations.

- The effect of the influenza A(H1N1)pdm09 virus on mortality

In their comment, the authors implied that the 2009 influenza A(H1N1) pandemic virus did not affect mortality in tropical climate countries. However, global mortality studies have shown that the 2009 influenza A(H1N1) pandemic virus did in fact affect mortality in tropical climate countries.^19^, ^20^ Both Dawood and Simonsen estimated high rates of influenza‐associated respiratory death related to the emergence of influenza A(H1N1)pdm09 in some tropical climate countries compared to other parts of the world.^19^, ^20^ Specifically, the influenza mortality estimated among <65 years old was higher in South‐East Asia during the pandemic year (2009) when compared with seasonal influenza epidemics.^20^ In addition, Alonso et al indicated that influenza A(H1N1)pdm09 did not affect mortality rates in Madagascar. However, a study by Rajatonirina et al,^21^ found an increase in the number of deaths during the weeks following the emergence of influenza A(H1N1)pdm09 in the country. Our article, however, did not focus on the pandemic virus, but on seasonal influenza viruses, which are likely to affect burden in populations differently than newly emerged influenza virus when population immunity in specific age groups may be limited. Therefore, our estimated influenza‐associated mortality of 6‐11 per 100 000 population per year in Bangladesh is a seasonal burden estimate, and we do not think that a comparison with the 2009 pandemic is valid in this context.

- Poor match in timing of respiratory deaths and influenza circulation

The authors note that there was dissociation of peaks of influenza virus circulation in the sentinel surveillance data and acute respiratory illness (ARI)‐associated mortality in the catchment areas in the months of December‐February in Bangladesh. We too observed this offset and considered it in our analytic approach to estimate influenza‐associated ARI deaths for Bangladesh. In our methods, we calculated the influenza‐associated mortality incidence using monthly ARI death counts and the monthly proportion of samples that tested positive for influenza viruses. As stated on page 67 of our article (the equation),^2^ the monthly mortality rates were derived by multiplying the proportion of ARI deaths (*D*/*P*) by the monthly proportion of influenza‐positive specimens (*F*) and then added for a consecutive 12‐month period to produce the annual estimates. If for a given month, the proportion of samples testing influenza positive was zero (ie *F *=* *0), ARI deaths (*D*/*P*) from that month would not be attributed to influenza nor contribute to the annual mortality rate estimates as multiplying the ARI deaths by 0% influenza positive (*D*/*P* * *F*) would also be zero. Thus, the estimates for Bangladesh did account for the offset between virus circulation and ARI mortality.

- Mortality or hospitalization attribution to other underlying conditions or pathogens

The authors also note their concern that we may be attributing influenza virus infections to deaths among persons with preexisting conditions or terminal illnesses. In the survey for all age groups in Bangladesh, the decedents must meet the case definitions as defined in the article and “the disease had to be a new onset and not a progression of a pre‐existing condition.^2^” [Page 67. section 2.3]. Therefore, we excluded those deaths where the respondents reported terminal illnesses and the ARI was not of new onset. We agree that there could still be misattribution of disease burden, but one must also consider that we focused our survey on respiratory disease burden, which represents only a portion of total influenza‐attributable mortality. Circulatory or other non‐respiratory deaths could also be attributed to influenza virus infections.^22^

Furthermore, we agree that RSV surveillance and burden estimates are very important to consider in these settings.^23^ To address this, an analysis exploring the effect of respiratory syncytial virus (RSV) circulation on ARI deaths among children <5 years of age in Bangladesh is underway and similar studies could be conducted in Rwanda. Nonetheless, the estimated influenza‐associated hospitalization rates among children aged <5 years in Rwanda (168 per 100 000 population)^3^ are similar to those of South Africa (156 per 100 000 population),^11^ other countries in Africa,^8^, ^9^, ^12^ and global estimates for Africa (174 per 100 000 population).^13^ Whereas the circulation patterns of RSV are poorly understood in Rwanda and other African countries, in South Africa, the RSV season precedes and minimally overlaps with the influenza season.^24^ In this situation, misattribution of influenza‐ vs. RSV‐associated hospitalization or mortality is unlikely to occur. Nonetheless, irrespective of RSV cocirculation, the attribution of an illness episode to a single pathogen or condition remains difficult.

- Comparison with IHME estimates

The authors also compared our estimates to those estimated by the Institute for Health Metrics and Evaluation (IHME). The IHME methods for estimating global influenza deaths focus on attributing influenza virus infections to lower respiratory tract infection deaths,^25^ while we focused on associating influenza to a wider case definition of acute respiratory infections. The IHME approach attributes a death to a single pathogen or condition as primary cause of death, which requires careful interpretation as some viral pathogens, such as influenza, have been observed to exacerbate some chronic medical conditions, such as asthma or chronic lung and cardiac conditions. Furthermore, more than one pathogen can be involved in the pathogenesis pathway of an illness episode. The methods used by IHME to estimate influenza‐associated deaths are different from what many influenza researchers utilize when they estimate mortality burden. A comparison of the IHME approach to other methods used to estimate global influenza deaths is further discussed in Iuliano et al^26^ Lastly, IHME mentions in their limitations that their estimates are not representative of non‐hospitalized disease burden,^25^ which we measured using community surveys to capture deaths occurring outside of hospitals. Thus, we would expect our estimates to be higher than the IHME estimates. In Bangladesh, especially in rural areas, healthcare utilization is very low^27^ and many deaths take place outside the health system.^28^ We therefore do not think IHME estimates for influenza death are comparable with our estimate.

We would like to thank the authors for their discussion of our papers.
